# Colossal figure of merit and compelling HER catalytic activity of holey graphyne

**DOI:** 10.1038/s41598-023-35016-8

**Published:** 2023-06-05

**Authors:** Muhammad Sajjad, Surabhi Suresh Nair, Yarjan Abdul Samad, Nirpendra Singh

**Affiliations:** 1 Department of Physics, Khalifa University of Science and Technology, 127788 Abu Dhabi, United Arab Emirates; 2 Department of Aerospace Engineering, Khalifa University of Science and Technology, 127788 Abu Dhabi, United Arab Emirates; 3 Cambridge Graphene Centre, Department of Engineering, University of Cambridge, Cambridge, UK

**Keywords:** Photocatalysis, Thermoelectrics

## Abstract

Herein, we have conducted a comprehensive study to uncover the thermal transport properties and hydrogen evolution reaction catalytic activity of recently synthesized holey graphyne. Our findings disclose that holey graphyne has a direct bandgap of 1.00 eV using the HSE06 exchange–correlation functional. The absence of imaginary phonon frequencies in the phonon dispersion ensures its dynamic stability. The formation energy of holey graphyne turns out to be − 8.46 eV/atom, comparable to graphene (− 9.22 eV/atom) and *h*-BN (− 8.80 eV/atom). At 300 K, the Seebeck coefficient is as high as 700 μV/K at a carrier concentration of 1 × 10^10^ cm^-2^. The predicted room temperature lattice thermal conductivity (κ_l_) of 29.3 W/mK is substantially lower than graphene (3000 W/mK) and fourfold smaller than C_3_N (128 W/mK). At around 335 nm thickness, the room temperature κ_l_ suppresses by 25%. The calculated *p*-type figure of merit (*ZT*) reaches a maximum of 1.50 at 300 K, higher than that of holey graphene (*ZT* = 1.13), γ-graphyne (*ZT* = 0.48), and pristine graphene (*ZT* = 0.55 × 10^–3^). It further scales up to 3.36 at 600 K. Such colossal *ZT* values make holey graphyne an appealing *p*-type thermoelectric material. Besides that, holey graphyne is a potential HER catalyst with a low overpotential of 0.20 eV, which further reduces to 0.03 eV at 2% compressive strain.

## Introduction

Rapidly growing population and infrastructure development are behind the rising energy demand, which will further increase from 23 Terawatts in 2030 to 30 Terawatts in 2050^1^. According to the Global Renewable Energy Community (REN21) statistics, nearly 80% of the total energy relies on conventional energy resources and renewable energy sources add only the rest 20%^2^. An excessive dependence on fossil fuels causes global warming and destructive environmental issues^3^. There has been a worldwide push to find sustainable and clean alternatives to fossil fuels to counter such problems^4^. Among the natural renewable energy sources, hydrogen is an ideal sustainable energy source due to its high energy density and environmentally benign^5^. However, precious and less abundant metal-based catalysts have been used for hydrogen production^6^, impeding their widespread utilization^7^. Therefore, exploring novel and metal-free catalysts is a viable route for the mass production of hydrogen^8,9^. Thermoelectric generators are also excellent alternatives for clean and renewable energy resources, considering the abundance of waste heat accompanied by infrequent maintenance and long device life, as no moving parts are involved in the technology^10,11^. Although Bi_2_Te_3_ has been widely used in thermoelectric generators, the toxicity and scarcity of tellurium restrict their usage^12^. In addition, bipolar conduction suppresses the figure of merit of Bi_2_Te_3_ above 450 K due to its narrow bandgap^12^. Hence abundant and non-toxic materials with reasonable bandgap would be an optimal choice.

Since the experimental realization of graphene^13^, tremendous attention has been devoted to other two-dimensional (2D) carbon allotropes due to their peculiar physical properties^14^, topological states^15^, massless Dirac cones^16,17^, and semiconducting behavior^18,19^. Porous nitrogenated holey graphene^20^, polyaniline^21^, phagraphene^22^, naphyne^23^, graphtetrayne^24^, and biphenylene^25^ are a few examples of experimentally synthesized 2D carbon allotropes.

Among these, graphyne, with varying *sp* and *sp*^*2*^ hybridized carbon atoms constitutes one of the biggest families of graphene allotropes^26^. They possess exceptional flexibility, high carrier mobility, a Dirac cone characterized electronic band structure, efficient adsorption of ions and molecular selectivity due to porous structures, and reduced thermal conductivity due to acetylenic bonds with *sp* state^27–32^. Recently, the bottom-up technique has been employed to synthesize an ultrathin 2D carbon allotrope named holey graphyne^33^. The nanosheet shows excellent mechanical, thermal, and dynamic stability. Unlike graphene, it is a direct bandgap semiconductor with high carrier mobility (promising for applications in optoelectronics) and possesses *sp* and *sp*^*2*^ hybridized carbon atoms uniformly distributing the porous architectures (favorable for gas separation, water desalination, energy storage, and catalysis)^34^. Holey graphyne may also be considered an anchoring material in metal-sulfur batteries, like other materials with similar crystal structures previously researched for this purpose^35,36^. However, to the best of our knowledge, none of these applications of holey graphyne has been uncovered so far. Herein, we conducted a comprehensive study to explore its potential in thermoelectricity and H_2_ production. By the presence of unique distinct bonding, κ_l_ is anticipated to be lower in holey graphyne compared to the other flat 2D materials from the graphene family, resulting in an enhanced thermoelectric figure of merit. On the other hand, variation in bond charge density accompanied by a highly porous plane, which increases the number of reactive sites, makes it an excellent choice for catalysis.

## Computational details

The Vienna Ab-initio Simulation Package (VASP)^37,38^ is used to perform the density functional theory (DFT) calculations by employing the Perdew − Burke − Ernzerhof and Heyd − Scuseria − Ernzerhof (HSE06) hybrid exchange–correlation functionals^39,40^. A plane wave cutoff energy of 550 eV and a Γ-centred *k*-mesh of 9 × 9 × 1 (30 × 30 × 1) is used to sample the first Brillouin zone for self-consistent (non-self-consistent) calculations. The crystal is optimized until Hellmann–Feynman forces drop below 10^–4^ eV/Å. A vacuum of 15 Å, perpendicular to the sheet, is taken to eliminate the interactions between adjacent layers. The structural formation energy is calculated using E_form._ = (E_tot._ − *n*.E_C_)/*n* formula, where E_tot._ and E_C_ are the total energies of holey graphyne and an isolated C atom, respectively, and *n* is the total number of atoms in a unit cell. The phonon dispersion, thermal transport coefficients, and lattice thermal conductivity are calculated using the Hiphive^41^, BoltzTraP2^42^, and the ShengBTE^43^ codes, respectively. This sophisticated method has been used to analyze the transport characteristics of numerous materials^44–46^. As inputs to ShengBTE, the 2nd and 3rd-order force constants are computed using a 3 × 3 × 1 supercell. The 3rd-order force constants are calculated by displacing atoms up to the tenth nearest neighbors. A dense *q*-mesh 30 × 30 × 1 is utilized to obtain well-converged lattice thermal conductivity. The produced lattice thermal conductivity is well converged, as the value at 300 K varies by less than 4% and 1% from the result obtained with displacing atoms up to the ninth nearest neighbors and 20 × 20 × 1 *q*-mesh, respectively. The change in Gibbs free energy change (ΔG_H_) is calculated using the relation, ΔG_H_ = ΔE_H_ + ΔE_ZPE_ − TΔS, where ΔE_H_, ΔE_zpe_, T, and ΔS stand for the hydrogen adsorption energy, change in the zero-point energy, temperature (298.15 K), and change in entropy, respectively^47^. The value of ΔE_ZPE_ – TΔS is equal to 0.24 eV, giving rise to ΔG_H_ = ΔE_H_ + 0.24^47^. The adsorption energy of hydrogen is obtained as ΔE_H_ = E_total_ − E_pristine_ − 1/2E_H2_, where E_total_, E_pristine_, and E_H2_ are the total energies of holey graphyne with adsorbed H atom, pristine holey graphyne, and single H_2_-molecule in the gas phase, respectively.

## Results and discussions

**Figure 1:**
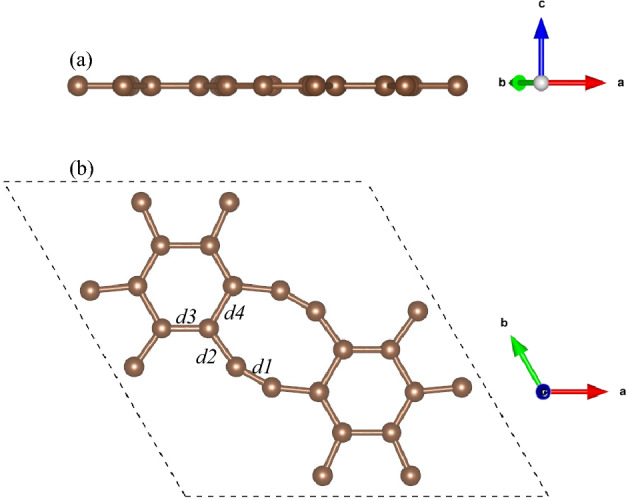
The side (**a**) and top (**b**) views of the optimized crystal structure of holey graphyne unit cell. di (i = 1–4) represents the bond length. Dashed lines enclose the unit cell.

**Figure 2:**
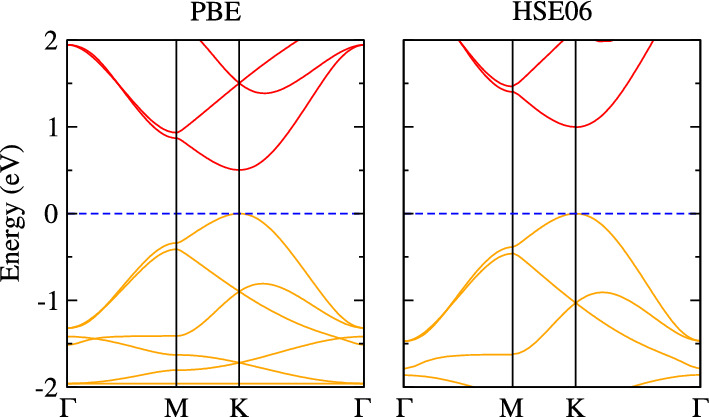
Calculated electronic band structures of holey graphyne. The orange and red curves correspond to valence and conduction bands, respectively. The blue dashed line represents the Fermi level.

Holey graphyne contains two rings comprised of six and eight carbon atoms, with a big pore, as shown in Fig. 1. Each primitive cell has an optimized in-plane lattice constant of 10.84 Å, in agreement with the previous study^33^. The C–C bonds have different bond lengths of 1.23 Å (*d1; sp* hybridized triple bond), 1.41 Å (*d2; sp* hybridized single bond), 1.40 Å (*d3; sp*^*2*^ hybridized double bond), and 1.46 Å (*d4; sp*^*2*^ hybridized single bond), as shown in Fig. 1b. The formation energy of holey graphyne is − 8.46 eV/atom, comparable to graphene (− 9.22 eV/atom) and *h*-BN (− 8.80 eV/atom) monolayers^48^. It is worthwhile to point out that holey graphyne has been experimentally synthesized using the bottom-up approach^33^. Besides that, *ab*-initio molecular dynamics simulations have determined that holey graphyne is thermally stable even at higher temperatures of 900 K.^33^ Fig. 2 shows the calculated direct bandgaps of 0.50 eV (PBE) and 1.0 eV (HSE06), which agrees with the previously calculated value and is also close to the experimental value of 1.10 eV.^33^.

**Figure 3:**
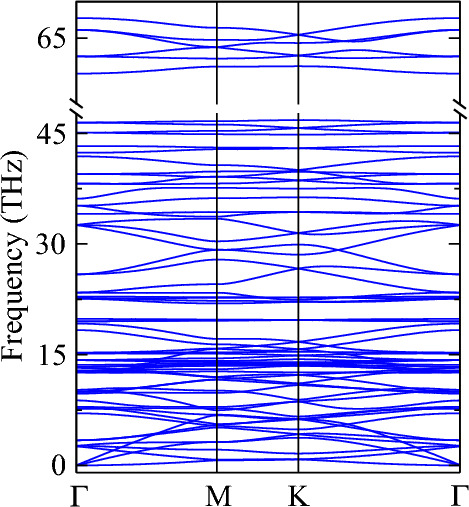
Calculated phonon dispersion of holey graphyne.

The calculated phonon dispersion of holey graphyne has a total of 72 phonon modes without imaginary frequencies, assuring its dynamic stability (see Fig. 3). The out-of-plane flexural acoustic (ZA) phonons have the lowest frequency among the acoustic modes, followed by the in-plane transverse acoustic (TA) and longitudinal acoustic (LA) phonon modes. The first optical phonon mode has a noticeably small frequency of 2.55 THz, favoring the high phonon–phonon scattering owing to the coupling between ZA and the lowest optical phonons favors high phonon–phonon scattering^49,50^. Also, the less dispersive nature of optical phonons results in their small phonon group velocities. These distinct characteristics collectively contribute to low κ_l_, compared to other carbon allotropes.

**Figure 4:**
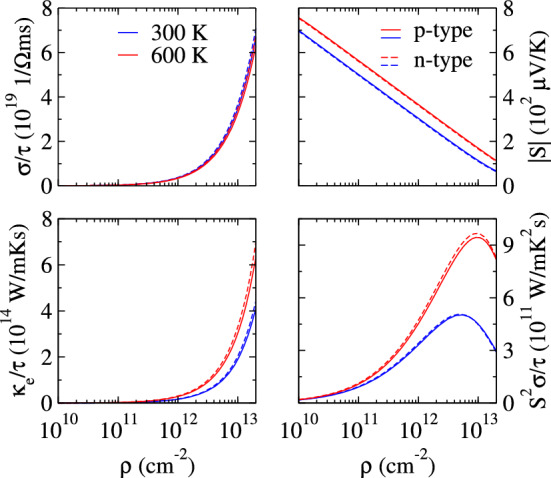
Calculated *p*-type and n-type electronic transport coefficients as a function of carrier concentration (ρ) at 300 K and 600 K.

Figure 4 reveals the electronic transport coefficients with respect to varying carrier concentrations and temperatures. The electronic part of electrical (σ/τ) and thermal (κ_e_/τ) conductivities increases and the Seebeck coefficient (*S*) declines linearly with carrier concentration^51^. The room temperature |*S*| (300 µV/K at 1 × 10^12^ cm^−2^) is approximately 6 times greater than that of graphene (~ 50 µV/K) at the same carrier concentration^52^. The *p*-type (*n*-type) |*S*| turns out to be 114 µV/K (110 µV/K) even at the highest considered carrier concentration (2 × 10^13^ cm^−2^) and at 600 K. The *p*-type S^2^σ/τ rises with growing carrier concentration until it approaches 5 × 10^11^ W/mK^2^s (9 × 10^11^ W/mK^2^s) at 300 K (600 K) and then drops as the doping concentration elevates. Such a remarkably high S and S^2^σ/τ suggest that investigating holey graphyne for thermoelectrics is worthwhile. It is further notable that identical dispersions of the valence band maxima and the conduction band minima result in similar variance in *p*-type and *n*-type electronic transport coefficients.

**Figure 5:**
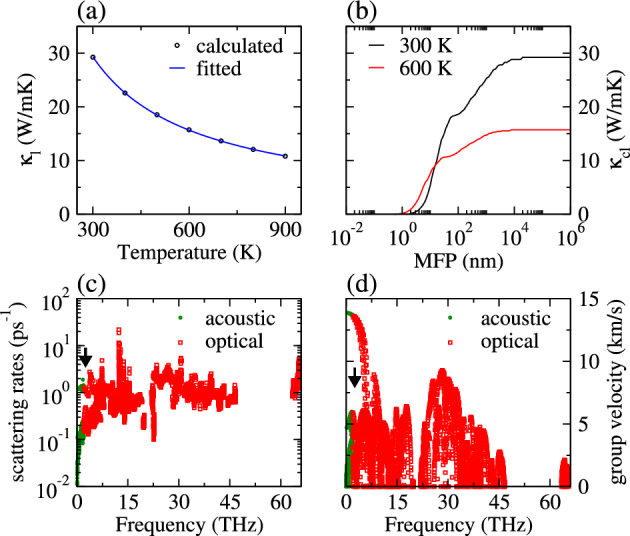
(**a**) Calculated and fitted (κ_l_ ∝ 1/T) κ_l_ as a function of temperature, (**b**) cumulative lattice thermal conductivity (κ_cl_) as a function of phonon mean free path (MFP), (**c**) phonon scattering rates, and (**d**) phonon group velocities at 300 K as a function of phonon frequency. The black arrows in (**c** and **d**) represent frequency 2.55 THz of first optical phonon mode at Γ point.

Further, we have looked into how lattice vibrations affect heat conduction in holey graphyne (see Fig. 5a). The calculated room temperature κ_l_ turns out to be 29.3 W/mK, lower than graphene (3000 W/mK)^53^, γ-graphyne (76.4 W/mK)^54^, C_3_N (128 W/mK)^53^, and C_2_N (82.22 W/mK)^55^. The calculated κ_l_ at 300 K differs by 0.01% from the results obtained using 20 × 20 × 1 *q*-mesh (i.e., 29.4 W/mK), leaving no relevant effect on our conclusion. The atom displacement to the ninth nearest neighbors yields κ_l_ of 28.05 W/mK, which deviates by 4% from the value obtained by displacing atoms up to the tenth nearest neighbors. The ultralow κ_l_ of holey graphyne, which is appealing from the thermoelectric perspective, is attributed to its bonds being less stiff than those of the above-mentioned flat materials. The C–C bond stiffness is determined by the spring constant (*K*) of holey graphyne, which is calculated as the trace of the harmonic force constant tensor between the closest adjacent atoms. It is written as $K={\Phi }_{CC}^{xx}+ {\Phi }_{CC}^{yy}+{\Phi }_{CC}^{zz}$, where ${\Phi }_{MX}^{\alpha \alpha }$ is the second derivate of energy with respect to displacement of atoms along the Cartesian axis α. The bond stiffness of *d1*, *d2*, *d3*, and *d4* are 87 eV/Å^2^, 36 eV/Å^2^, 44 eV/Å^2^, and 31 eV/Å^2^, which are substantially smaller than that of graphene (10,105 eV/Å^2^). Such a bond feature inhibits heat transfer via lattice vibrations, leading to low κ_l_ values. Figure 5a shows that as temperature increases κ_l_ decreases due to the pronounced phonon–phonon scattering and follows the relation κ_l_ ∝ 1/T.

Nanostructuring of materials, where materials are composed of nanometer size grains and further with nanoscale internal structures^56^, can reduce κ_l_ without affecting σ. To better comprehend the scope of phonon engineering, the cumulative lattice thermal conductivity (κ_cl_) as a function of the phonon mean free path (MFP) is investigated and presented in Fig. 5b. As MFP decreases, the phonons scattering increases reducing the heat transfer. The contribution of phonons with different MFPs to lattice thermal conductivity is studied by calculating κ_cl_, thereby deducing phonons most relevant to thermal conduction^43^. At 300 K (600 K) 75% of κ_l_ is by phonons having MFP 335 nm (110 nm), which implies reducing κ_l_ through nanostructuring is a viable strategy for holey graphyne. In other words, a sample size of 335 nm (110 nm) could help reduce the inherited value of κ_l_ at 300 K (600 K) by one-fourth. κ_cl_ increases (decreases) with rising MFP (temperature) and shows a plateau above 20,092 nm (7924 nm) at 300 K (600 K). A lower plateau at 600 K is due to the stronger phonon scattering at elevated temperatures^10^. Given that phonon transports are predominantly dependent on phonon scattering rates and phonon group velocities, our calculated results for acoustic and optical phonon modes are illustrated in Fig. 5c,d. The highest scattering rate for acoustic phonons at room temperature is 2.74 ps^−1^, comparatively higher than C_3_N monolayer (2 ps^−1^)^53^. It is due to the coupling of acoustic and optical phonon modes, which leads to increased scattering rates of acoustic phonons, leading to a considerable reduction of κ_l_^50^. The phonon group velocities are calculated to validate the above analysis and presented in Fig. 5d. The highest phonon group velocity of acoustic phonon mode at room temperature is 13.9 km/s, much lower than that of graphene (~ 22 km/s)^57^ and nitrogenated holey graphene (18.48 km/s)^58^. Such low group velocity is a consequence of flat phonon modes (see Fig. 3)^50^. Thus, smaller κ_l_ of holey graphyne arises from low phonon group velocity and high scattering rates.

**Figure 6:**
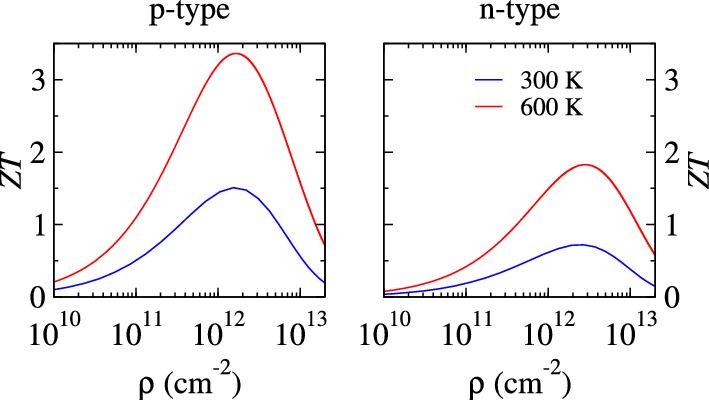
Calculated figure of merit (ZT) of holey graphyne.

The figure of merit (*ZT*) depends on the relaxation time (τ) and is vital for showcasing the potential of a material for its thermoelectric applications. In this work, we have adapted the value for τ from the deformation potential theory used in the previous study^33^. The relation τ_T_ = 300*τ_300_/T is engaged to yield its value at 600 K. The values of τ for holes (electrons) turn out to be 3.27 ps (1.16 ps) and 1.64 ps (0.58 ps) at 300 K and 600 K, respectively. The calculated *ZT* in Fig. 6 follows an upward trend similar to S^2^σ/τ. The *p*-type *ZT* is higher than the *n*-type and is counter to the trend seen in electronic transport coefficients (see Fig. 4). It is a result of the fact that holes and electrons have different relaxation times. The relation *ZT* = S^2^T/(κ_e_/σ + κ_l_/σ), where κ_l_/σ is influenced by τ, explains that larger τ values result in greater *ZT*. The *ZT* secures peak values of 3.36 (1.50) and 1.82 (0.71) at 600 K (300 K) for *p*-type and *n*-type dopings, respectively. In the previous study^33^, *ab*-initio molecular dynamics simulations determined that holey graphyne is thermally stable even at higher temperatures of 900 K, as established based on the ab initio molecular dynamics simulations. The room temperature *p*-type *ZT* of holey graphyne is higher than that of holey graphene (1.13)^59^ and significantly higher than pristine graphene (0.55 × 10^–3^)^60^ and γ-graphyne (0.48)^54^.

**Figure 7:**
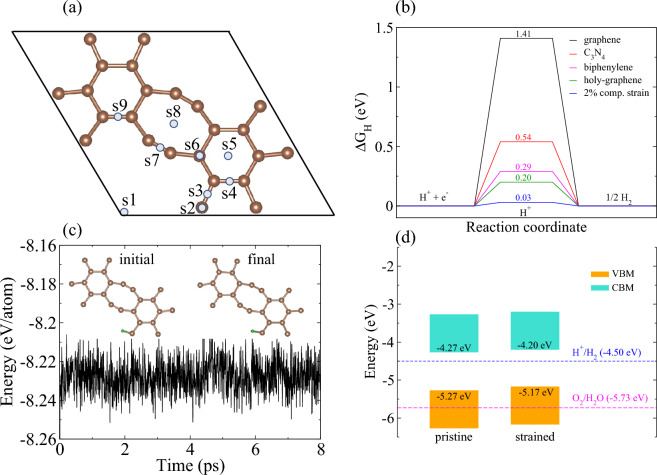
(**a**) The unit cell of holey graphyne with the possible adsorption sites (s1–s9) of hydrogen. (**b**) Calculated Gibbs free energy (ΔG_H_) of hydrogen adsorption on holey graphyne. The corresponding values on graphene [Ref. ^61^], C_3_N_4_ [Ref. ^63^], and biphenylene [Ref. ^61^] are also included for comparison. (**c**) The fluctuation of total energy during ab-initio molecular dynamics simulations at 300 K alongside the initial and the final hydrogen-adsorbed holey graphene structures. The brown and green spheres represent C and H atoms, respectively. (**d**) Calculated band edge positions of pristine and strained (2% compressive strain) holey graphene relative to the vacuum level.

Since active and cost-effective metal-free catalysts, as opposed to costly Pt-based catalysts, for HER are necessary to attain sustainable energy systems. We have also uncovered the response of holey graphyne to HER. As the standard descriptor for HER activity is the Gibbs free energy (ΔG_H_), which is derived from the hydrogen adsorption energy. In the first instance, the H atom is adsorbed on all the possible 9 adsorption sites (s1–s9 in Fig. 7a). The H atom finds the s2 site the most favorable. The corresponding ΔG_H_ of s2 site is 0.20 eV, which is much smaller than that of graphene (1.41 eV)^61^, phosphorene (1.25 eV)^62^, and C_3_N_4_ (0.58 eV)^63^ and comparable to biphenylene (0.29 eV)^61^, see Fig. 7b. As a matter of fact, ΔG_H_ = 0 stands out as an optimal value for HER. However, a value of |ΔG_H_|< 0.2 eV signifies the better catalytic performance of materials for HER activity^64^. We further engaged strain engineering to evaluate its impact on ΔG_H_. It is observed that 2% compressive strain improves the catalytic performance of holey graphyne by reducing ΔG_H_ to 0.03 eV. Moreover, the-ab initio molecular dynamics simulation assures the thermal stability of hydrogen adsorbed holey graphyne concerning the low energy fluctuations alongside the intact H and the sheet structure after a time of 8 ps (see Fig. 7c). To address the photocatalytic performance of holey graphyne, the positions of valence band maxima (VBM) and conduction band minima (CBM) relative to the vacuum level along with H_+_/H_2_ reduction and (O_2_/H_2_O) oxidation potentials for water splitting are presented in Fig. 7d. It is evident that CBM is more positive than the H^+^/H_2_ potential, suggesting that holey graphyne is a potential material for photocatalytic hydrogen production. In contrast, the VBM is higher than the O_2_/H_2_O oxidation potential; hence, the holey graphene is unsuitable for oxidizing H_2_O to O_2_. However, an appropriate band engineering to shift the VBM downwards (e.g., doping, application of an external bias, heterojunction, etc.) may enable the water splitting into H_2_ and O_2_.

## Conclusions

Using the first-principles computations, we investigated the thermal transport properties and HER catalytic activity of holey graphyne, a semiconducting material with a direct bandgap of 1.0 eV. The closely packed phonons display positive frequencies across the entire Brillouin zone, assuring the dynamic stability of holey graphyne. The room temperature |S| is as high as 300 μV/K at 1 × 10^12^ cm^−2^ (nearly sixfold than graphene). Flexural acoustic phonons couple with the lowest optical phonon mode to enhance phonon–phonon scattering and eventually decrease κ_l_. The estimated room temperature κ_l_ of 29.3 W/mK is ultralow compared to that of graphene (3000 W/mK), attributed to its low bond stiffness. With the aid of nanostructure engineering, κ_l_ further reduces by a factor of four for a crystal size of around 335 nm (110 nm) at 300 K (600 K). The room temperature *p*-type *ZT* achieves a maximum value of 1.50, largely greater than graphene (0.55 × 10^–3^), demonstrating its superior thermoelectric performance. Lastly, holey graphyne having low overpotential and more positive CBM than the H^+^/H_2_ potential, is also capable of catalyzing HER.

## Data Availability

The datasets used and/or analysed during the current study available from the corresponding author on reasonable request.

## References

[1] Lewis NS, Nocera DG (2006). Powering the planet: Chemical challenges in solar energy utilization. Proc. Natl. Acad. Sci..

[2] GSR2018_Full-Report_English.

[3] Kverndokk, S. *Depletion of Fossil Fuels and the Impact of Global Warming*; Statistics Norway, Research Department: Oslo (1994). http://hdl.handle.net/10419/192091.

[4] Kabeyi MJB, Olanrewaju OA (2022). Sustainable energy transition for renewable and low carbon grid electricity generation and supply. Front. Energy Res..

[5] Dunn S (2002). Hydrogen futures: Toward a sustainable energy system. Int J. Hydrog. Energy.

[6] Wu H-H, Huang H, Zhong J, Yu S, Zhang Q, Zeng XC (2019). Monolayer triphosphates MP _3_ (M = Sn, Ge) with excellent basal catalytic activity for hydrogen evolution reaction. Nanoscale.

[7] Zhang J, Sasaki K, Sutter E, Adzic RR (2007). Stabilization of platinum oxygen-reduction electrocatalysts using gold clusters. Science (1979).

[8] Tymoczko J, Calle-Vallejo F, Schuhmann W, Bandarenka AS (2016). Making the hydrogen evolution reaction in polymer electrolyte membrane electrolysers even faster. Nat. Commun..

[9] Sahoo MR, Ray A, Singh N (2022). Theoretical insights into the hydrogen evolution reaction on VGe _2_ N _4_ and NbGe _2_ N _4_ monolayers. ACS Omega.

[10] *Recent Trends in Thermoelectric Materials Research II*; Semiconductors and semimetals ; v. 70; Academic Press: San Diego (2001).

[11] Snyder GJ, Toberer ES (2008). Complex thermoelectric materials. Nat. Mater..

[12] Pei J, Cai B, Zhuang H-L, Li J-F (2020). Bi2Te3-based applied thermoelectric materials: Research advances and new challenges. Natl. Sci. Rev..

[13] Novoselov KS, Geim AK, Morozov SV, Jiang D, Zhang Y, Dubonos SV, Grigorieva IV, Firsov AA (2004). Electric field effect in atomically thin carbon films. Science (1979).

[14] Allen MJ, Tung VC, Kaner RB (2010). Honeycomb carbon: A review of graphene. Chem. Rev..

[15] Zhao M, Dong W, Wang A (2013). Two-dimensional carbon topological insulators superior to graphene. Sci. Rep..

[16] Malko D, Neiss C, Viñes F, Görling A (2012). Competition for graphene: Graphynes with direction-dependent dirac cones. Phys. Rev. Lett..

[17] Zhang X, Wei L, Tan J, Zhao M (2016). Prediction of an ultrasoft graphene allotrope with dirac cones. Carbon N. Y..

[18] Jiang J-W, Leng J, Li J, Guo Z, Chang T, Guo X, Zhang T (2017). Twin graphene: A novel two-dimensional semiconducting carbon allotrope. Carbon N. Y..

[19] Zhang W, Chai C, Fan Q, Song Y, Yang Y (2021). Two-dimensional carbon allotropes with tunable direct band gaps and high carrier mobility. Appl. Surf Sci..

[20] Mahmood J, Lee EK, Jung M, Shin D, Jeon IY, Jung SM, Choi HJ, Seo JM, Bae SY, Sohn SD, Park N, Oh JH, Shin HJ, Baek JB (2015). Nitrogenated holey two-dimensional structures. Nat. Commun..

[21] Mahmood J, Lee EK, Jung M, Shin D, Choi H-J, Seo J-M, Jung S-M, Kim D, Li F, Lah MS, Park N, Shin H-J, Oh JH, Baek J-B (2016). Two-dimensional polyaniline (C 3 N) from carbonized organic single crystals in solid state. Proc. Natl. Acad. Sci..

[22] Fan Q, Martin-Jimenez D, Ebeling D, Krug CK, Brechmann L, Kohlmeyer C, Hilt G, Hieringer W, Schirmeisen A, Gottfried JM (2019). Nanoribbons with nonalternant topology from fusion of polyazulene: Carbon allotropes beyond graphene. J. Am. Chem. Soc..

[23] Li Y, Li Y, Lin P, Gu J, He X, Yu M, Wang X, Liu C, Li C (2020). Architecture and electrochemical performance of alkynyl-linked naphthyl carbon skeleton: Naphyne. ACS Appl. Mater. Interfaces.

[24] Pan Q, Chen S, Wu C, Shao F, Sun J, Sun L, Zhang Z, Man Y, Li Z, He L, Zhao Y (2021). Direct synthesis of crystalline graphtetrayne—a new graphyne allotrope. CCS Chem..

[25] Fan Q, Yan L, Tripp MW, Krejčí O, Dimosthenous S, Kachel SR, Chen M, Foster AS, Koert U, Liljeroth P, Gottfried JM (2021). Biphenylene network: A nonbenzenoid carbon allotrope. Science (1979).

[26] Kim BG, Choi HJ (2012). Graphyne: Hexagonal network of carbon with versatile dirac cones. Phys Rev B.

[27] Ouyang T, Cui C, Shi X, He C, Li J, Zhang C, Tang C, Zhong J (2020). Systematic enumeration of low-energy graphyne allotropes based on a coordination-constrained searching strategy. Phys. Status Solidi (RRL) Rapid Res. Lett..

[28] Kou J, Zhou X, Lu H, Wu F, Fan J (2014). Graphyne as the membrane for water desalination. Nanoscale.

[29] Yan P, Ouyang T, He C, Li J, Zhang C, Tang C, Zhong J (2021). Newly discovered graphyne allotrope with rare and robust dirac node loop. Nanoscale.

[30] Ouyang T, Xiao H, Xie Y, Wei X, Chen Y, Zhong J (2013). Thermoelectric properties of gamma-graphyne nanoribbons and nanojunctions. J. Appl. Phys..

[31] Panigrahi P, Sajjad M, Singh D, Hussain T, Andreas Larsson J, Ahuja R, Singh N (2021). Two-dimensional nitrogenated holey graphene (C2N) monolayer based glucose sensor for diabetes mellitus. Appl. Surf. Sci..

[32] Sajjad M, Hussain T, Singh N, Larsson JA (2020). Superior anchoring of sodium polysulfides to the polar C2N 2D material: A potential electrode enhancer in sodium-sulfur batteries. Langmuir.

[33] Liu X, Cho SM, Lin S, Chen Z, Choi W, Kim Y-M, Yun E, Baek EH, Ryu DH, Lee H (2022). Constructing two-dimensional holey graphyne with unusual annulative π-extension. Matter.

[34] James A, John C, Owais C, Myakala SN, Chandra Shekar S, Choudhuri JR, Swathi RS (2018). Graphynes: Indispensable nanoporous architectures in carbon flatland. RSC Adv..

[35] Al-Jayyousi H, Eswaran MK, Ray A, Sajjad M, Larsson JA, Singh N (2022). Exploring the superior anchoring performance of the two-dimensional nanosheets B_2_C_4_P_2_ and B_3_C_2_P_3_ for lithium-sulfur batteries. ACS Omega.

[36] Al-Jayyousi HK, Sajjad M, Liao K, Singh N (2022). Two-dimensional biphenylene: A promising anchoring material for lithium-sulfur batteries. Sci. Rep..

[37] Kresse G, Furthmüller J (1996). Efficiency of Ab-initio total energy calculations for metals and semiconductors using a plane-wave basis set. Comput. Mater. Sci..

[38] Kresse G, Furthmüller J (1996). Efficient iterative schemes for Ab initio total-energy calculations using a plane-wave basis set. Phys. Rev. B.

[39] Perdew JP, Burke K, Ernzerhof M (1996). Generalized gradient approximation made simple. Phys. Rev. Lett..

[40] Krukau AV, Vydrov OA, Izmaylov AF, Scuseria GE (2006). Influence of the exchange screening parameter on the performance of screened hybrid functionals. J. Chem. Phys..

[41] Eriksson F, Fransson E, Erhart P (2019). The hiphive package for the extraction of high-order force constants by machine learning. Adv. Theory Simul..

[42] Madsen GKH, Carrete J, Verstraete MJ (2018). BoltzTraP2, a program for interpolating band structures and calculating semi-classical transport coefficients. Comput. Phys. Commun..

[43] Li W, Carrete J, Katcho A, Mingo N (2014). ShengBTE: A solver of the boltzmann transport equation for phonons. Comput. Phys. Commun..

[44] Wang B, Yan X, Cui X, Cai Y (2022). First-principles study of the phonon lifetime and low lattice thermal conductivity of monolayer γ-GeSe: A comparative study. ACS Appl. Nano Mater..

[45] Shu Z, Wang B, Cui X, Yan X, Yan H, Jia H, Cai Y (2023). High-performance thermoelectric monolayer γ-GeSe and its group-IV monochalcogenide isostructural family. Chem. Eng. J..

[46] Sajjad M, Singh N (2021). The impact of electron-phonon coupling on the figure of merit of Nb_2_SiTe_4_ and Nb_2_GeTe_4_ ternary monolayers. Phys. Chem. Chem. Phys..

[47] Nørskov JK, Bligaard T, Logadottir A, Kitchin JR, Chen JG, Pandelov S, Stimming U (2005). Trends in the exchange current for hydrogen evolution. J. Electrochem. Soc..

[48] Mortazavi B, Shojaei F, Yagmurcukardes M, Shapeev AV, Zhuang X (2022). Anisotropic and outstanding mechanical, thermal conduction, optical, and piezoelectric responses in a novel semiconducting BCN monolayer confirmed by first-principles and machine learning. Carbon N. Y..

[49] Mann S, Mudahar I, Sharma H, Jindal VK, Dubey GS, Gumbs G, Fessatidis V (2020). Lattice thermal conductivity of pure and doped (B, N) Graphene. Mater. Res. Express.

[50] Morelli DT, Slack GA (2006). High lattice thermal conductivity solids. High Therm. Conduct. Mater..

[51] Li L, Meller G, Kosina H (2007). Analytical conductivity model for doped organic semiconductors. J. Appl. Phys..

[52] Ali M, Pi X, Liu Y, Yang D (2018). Electronic and thermoelectric properties of atomically thin C_3_Si_3_/C and C_3_Ge_3_/C superlattices. Nanotechnology.

[53] Kumar S, Sharma S, Babar V, Schwingenschlögl U (2017). Ultralow lattice thermal conductivity in monolayer C3N as compared to graphene. J. Mater. Chem. A Mater..

[54] Jiang PH, Liu HJ, Cheng L, Fan DD, Zhang J, Wei J, Liang JH, Shi J (2017). Thermoelectric properties of γ-graphyne from first-principles calculations. Carbon N. Y..

[55] Ouyang T, Xiao H, Tang C, Zhang X, Hu M, Zhong J (2017). First-principles study of thermal transport in nitrogenated holey graphene. Nanotechnology.

[56] Singh DJ, Terasaki I (2008). Thermoelectrics: Nanostructuring and more. Nat. Mater..

[57] Mortazavi B, Podryabinkin EV, Novikov IS, Roche S, Rabczuk T, Zhuang X, Shapeev AV (2020). Efficient machine-learning based interatomic potentialsfor exploring thermal conductivity in two-dimensional materials. J. Phys. Mater..

[58] Zhao Y, Dai Z, Lian C, Meng S (2017). Exotic thermoelectric behavior in nitrogenated holey graphene. RSC Adv..

[59] Singh D, Shukla V, Ahuja R (2020). Optical excitations and thermoelectric properties of two-dimensional holey graphene. Phys. Rev. B.

[60] Anno Y, Imakita Y, Takei K, Akita S, Arie T (2017). Enhancement of graphene thermoelectric performance through defect engineering. 2d Mater..

[61] Luo Y, Ren C, Xu Y, Yu J, Wang S, Sun M (2021). A first principles investigation on the structural, mechanical, electronic, and catalytic properties of biphenylene. Sci. Rep..

[62] Cai Y, Gao J, Chen S, Ke Q, Zhang G, Zhang Y-W (2019). Design of phosphorene for hydrogen evolution performance comparable to platinum. Chem. Mater..

[63] Zheng Y, Jiao Y, Zhu Y, Li LH, Han Y, Chen Y, Du A, Jaroniec M, Qiao SZ (2014). Hydrogen evolution by a metal-free electrocatalyst. Nat. Commun..

[64] Huang B, Zhou N, Chen X, Ong W, Li N (2018). Insights into the electrocatalytic hydrogen evolution reaction mechanism on two-dimensional transition-metal carbonitrides (MXene). Chem. A Eur. J..

